# The Prediabetes Outcome at National Guard Primary Health Care Centers in Riyadh, Saudi Arabia: Retrospective Chart Review

**DOI:** 10.7759/cureus.10227

**Published:** 2020-09-03

**Authors:** Mohammed A Alateeq, Moath Aljohani, Sondos S Kinani, Ibrahim A Aljabr, Abdullah A Alduayji, Abdulrhman Aloud, Elham Alzahrani, Khalid Alharbi

**Affiliations:** 1 Family Medicine, Ministry of National Guard-Health Affairs, King Saud Bin Abdulaziz University for Health Sciences, Riyadh, SAU; 2 Family and Community Medicine, College of Medicine and Medical Sciences, Qassim University, Qassim, SAU; 3 Family Medicine, College of Medicine, King Saud Bin Abdulaziz University for Health Sciences, Riyadh, SAU; 4 Family Medicine, College of Medicine and Medical Sciences, Qassim University, Qassim, SAU

**Keywords:** outcome, primary care, metformin, prediabetes

## Abstract

Objectives

To identify the outcome of prediabetes and the interventions that have been implemented for prediabetic patients at primary healthcare centers (PHCs) affiliated with King Abdulaziz Medical City, Riyadh, Saudi Arabia.

Methodology

This retrospective chart-review study was carried out using the BestCare electronic health records (EHRs) system. Data from the PHCs of King Abdulaziz Medical City, Riyadh, Saudi Arabia were extracted. Inclusion criteria were patients with prediabetes who were diagnosed between January 2015 and December 2016, with at least one follow-up visit. Variables included demographics, comorbidities, blood sugar lab results, and lipid profile measurements at each visit and intervention at the time of the initial diagnosis. Fisher's Exact test, sign test, and Kruskal-Wallis test were used to assess the differences for non-normally-distributed variables, while a paired t-test was conducted for paired and normally distributed continuous variables. Data were analyzed using the statistical program SAS, version 9.4 (SAS Institute Inc. Cary, NC).

Result

Of the 92 patients followed up with for three years, 76.08% remained in the prediabetic range, while 16.4% regressed to a normal glycemic state (NGS) and 7.6% progressed to the diabetic range after intervention and follow-up for three years. Metformin use was not significant in the glycemic outcome. In comparison to the baseline, there was a considerable reduction in fasting blood sugar (FBS) and glycosylated hemoglobulin A1c (HbA1c) at the end of the follow-up.

Conclusion

We found that most of the patients remained in the prediabetic range after the three-year follow-up, with or without intervention. A commonly prescribed pharmacological intervention like metformin showed no regression benefit in most patients. More extensive prospective studies are needed to evaluate the outcome and adherence to different interventions.

## Introduction

Prediabetes is a serious health condition in which the glucose level is elevated but not enough for a diagnosis of diabetes [1]. The American Diabetic Association (ADA) defines prediabetes as fasting blood sugar (FBS) of 100-125 mg/dL (5.6-6.9 mmol/L), impaired glucose tolerance (IGT) ﻿2-hour plasma glucose (2-h PG) during 75-g oral glucose tolerance test (OGTT) of 140 mg/dL (7.8 mmol/L) to 199 mg/dL (11.0 mmol/L), or glycosylated hemoglobulin A1c (HbA1c) of 5.7%-6.4% (39-47 mmol/mol) [2].

In Saudi Arabia, 25.5% of the population aged ≥30 years is living with prediabetes, placing more than three million people at risk for diabetes mellitus (DM) [3,4]. An alarming figure is that 5%-10% of prediabetic people annually progress to type 2 diabetes mellitus (T2DM); in addition, 40.3% of diabetic patients are unaware that they have the disease [3,5]. Studies showed an increased risk of cardiovascular disease and all-cause mortality among prediabetics [6]. Additionally, progression to DM poses a significant threat to the health care budget in Saudi Arabia, as DM accounts for 13.9% of total health expenditure [4]. Therefore, the study of prediabetes outcomes aligns with the Saudi national transformation plan’s strategic goals to increase efficiency and promote the prevention of health risks [7]. For that, a multi-dimensional control program/intervention approach is urgently needed to reverse the burden of diabetes in Saudi Arabia [4].

While different treatment modalities for DM exist, prevention is still the cornerstone of slowing down the escalating burden of diabetes [2]. Fortunately, 80% of cases of T2DM are preventable by simple interventions [4]. Recommended interventions include lifestyle interventions, which include 150 min/week of physical activity and a dietary recommendation to achieve 7% weight loss and improve insulin sensitivity [8]. A diabetes prevention program (DPP) trial provided the most robust evidence that a significant lifestyle modification could decrease the incidence of T2DM by 58% over the course of three years [9].On the other hand, pharmacological interventions are recommended for particularly high-risk groups; the most common intervention is metformin, which showed similar effectiveness in an Indian diabetic prevention study [10].

Identifying the outcome and intervention undertaken for prediabetics is useful for planning and implementing effective diabetes prevention programs and decreasing the burden of diabetes around the country [11,12]. Many studies have assessed the status of prediabetes in the Saudi population. Most of these studies focused on the prevalence and risk factors of diabetes [12,13]. There is a paucity of studies that focus on prediabetes outcomes and effective intervention after a follow-up period using retrospective or prospective data. Therefore, we conducted this multicenter retrospective chart review in primary health care centers (PHCs) affiliated with King Abdulaziz Medical City, National Guard, Riyadh to identify the outcome of prediabetes in the actual setting. Furthermore, we determined which intervention was taken to slow the progression to DM.

## Materials and methods

Study design and setting

This retrospective chart review was conducted to identify the outcome of prediabetes and the documented interventions for prediabetic patients in the National Guard PHCs from the beginning of January 2015 to the end of December 2016, with a follow-up period of three years. The centers included the ambulatory care center (ACC), King Abdulaziz City Housing (Iskan Al Yarmouk), the National Guard Comprehensive Specialized Clinic (NGCSC), the Health Care Specialty Center (HCSC), the Employee Health Clinic (EHC), King Saud City Housing (Dirab clinics), and the King Abdullah Specialist Children’s Hospital primary care clinic. This study was reviewed and approved by the Institutional Review Board (IRB) of the King Abdullah International Medical Research Center (KAIMRC). The confidentiality of data was guaranteed, and data were not disclosed unless for study purposes.

Participants

The inclusion criteria included all EHRs at National Guard PHCs in Riyadh for adults who were diagnosed with prediabetes as recommended by the ADA guidelines, i.e., FBS from 5.6 mmol/L to 6.9 mmol/L and HbA1c between 5.7%-6.4% [2], diagnosed in the period between January 1, 2015 and December 31, 2016 and has had at least one follow-up visit after the initial visit. The exclusion criteria were a diagnosis of DM or a normal glycemic state. 

Data collection method

Two trained data collectors extracted data from EHRs in the Best Care system at National Guard PHCs. The baseline characteristics of the data included: sociodemographic data (age, sex), body mass index (BMI), smoking status, medication history at baseline, hypertension (by diagnosis or current use of antihypertensive medication), history of gestational diabetes, polycystic ovary syndrome, dyslipidemia (by diagnosis or on lipid-lowering agents), and cardiovascular disease (CVD). Blood sample results at the time of diagnosis and on each visit included the lipid profile in serum (total cholesterol [TC], low-density lipoprotein cholesterol [LDL], high-density lipoprotein cholesterol [HDL], and triglyceride [TG]) and blood sugar results (FBS, OGTT, and HbA1c). FBS, HbA1c, and the lipid profile were measured by auto-analyzers at the in-house laboratory of each local primary health care center. Data related to interventions like metformin, referral to bariatric surgery, and referral to the dietician at baseline were also included. Because of the nature of the study design, lifestyle changes implemented by patients such as exercise level or dietary changes were not tracked.

Follow-up

The study included only those individuals who were available for follow-up testing. Patients were followed up with from the time of initial diagnosis to the last available visit with available testing. Patients had variable frequency of follow-up visits depending on their medical status and presence of other comorbidities as assessed by the treating physician. If the patient had two initial abnormal tests, i.e., FBS and HBa1c, the patient was followed up based on their last recorded test. If only a single test had one abnormal result, i.e., FBS or HbA1c alone, the patient was followed up based on that abnormal tests.

Outcome assessment

Outcome assessment was judged based on the patient’s last available blood glucose measurement according to ADA guidelines [2]. A patient was considered prediabetic if their ﻿FBS was 100 mg/dL (5.6 mmol/L) to 125 mg/dL (6.9 mmol/L), if their IGT2-h PG during 75-g OGTT was 140 mg/dL (7.8 mmol/L) to 199 mg/dL (11.0 mmol/L) or if their glycosylated HbA1c was 5.7%-6.4% (39-47 mmol/mol). If the patient’s last readings were above the aforementioned, the patient was considered to be in the diabetic range. If the patient’s readings were less, the patient was considered to be in the normal glycemic state (NGS).

Statistical analysis

Data were analyzed using the statistical program SAS (version 9.4, SAS Institute Inc., Cary, NC). Descriptive statistics were used to describe the study population at the baseline. Continuous variables were presented as mean and standard deviation (SD) or as median (IQR). Frequencies and proportions were calculated for categorical variables (gender, smoking, comorbidities, and type of intervention). Fisher's Exact test was used to determine associations between categorical variables, while the Kruskal-Wallis test was used for non-normally distributed continuous variables. A sign test was used to compare non-normally distributed continuous variables and the paired t-test with normally distributed continuous variables. Statistical tests were considered significant at p <0.05.

## Results

In total, 102 participants were extracted from BestCare EHR. Ten of them were excluded: five had diabetes at the baseline, three were followed up with at the hospital rather than at PHCs, and two had no follow-up visits. The other 92 were followed up with as early as January 2015 to December 2019 until the last recorded visit. Table 1 shows that the mean age of the participants at the time of recruitment was 48.32±11.23 years. Females constituted more than half of the sample (54.35%). Overall, participants were either overweight or obese (31.9±5.89). Co-morbidities such as hypertension (61.96%) and dyslipidemia (82.61%) were prevalent among study individuals. Initial FBS was 6±0.55, while HbA1c was 6.08±0.47. The most common intervention undertaken was metformin, though one patient was already using metformin at baseline. None of the participants were prescribed another antidiabetic medication such as glucagon-like-peptide-1 receptor agonist (GLP-1) and sulfonylurea. Other demographics and clinical characteristics of participants are shown in Table 1.

**Table 1:** Demographics and baseline clinical characteristics (n=92) Values are presented as mean ± standard deviation (SD), or numbers and percentages (%). BMI: body mass index. GDM: gestational diabetes mellitus, PCOS: polycystic ovarian syndrome, CVD: cardiovascular disease, FBS: fasting blood sugar, HbA1c:nhemoglobin A1c, LDL: low-density lipoprotein cholesterol, HDL: high-density lipoprotein cholesterol.

Variable	N	(%)
Age (mean ± standard deviation (SD))	48.3±11.2	
Gender		
Male	42	45.6
Female	50	54.3
BMI initial (mean ± standard deviation (SD))	31.9±5.89	
Smoking		
No	84	91.3
Yes	8	8.7
Hypertension		
No	35	38.0
Yes	57	61.9
History of GDM,(women, n=50).		
No	50	100
Yes	0	0
PCOS (women, n=50).		
No	46	95.6
Yes	4	4.3
Dyslipidemia		
No	16	17.3
Yes	76	82.6
CVD		
No	84	91.3
Yes	8	8.7
Lab readings on Initial visit:		
FBS (mmol/L)	6.0±0.55	
HbA1c ( %)	6.08±0.47	
LDL (mmol/L)	2.83±0.81	
Triglyceride (mmol/L)	1.43±0.62	
HDL (mmol/L)	1.16±0.46	
Total-cholesterol (mmol/L)	4.62±0.86	
Intervention:		
Metformin		
No	33	35.8
Yes	59	64.1
Referral to the bariatric surgery		
No	88	95.6
Yes	4	4.3
Referral to dietician		
No	78	84.7
Yes	14	15.2

**Figure 1:**
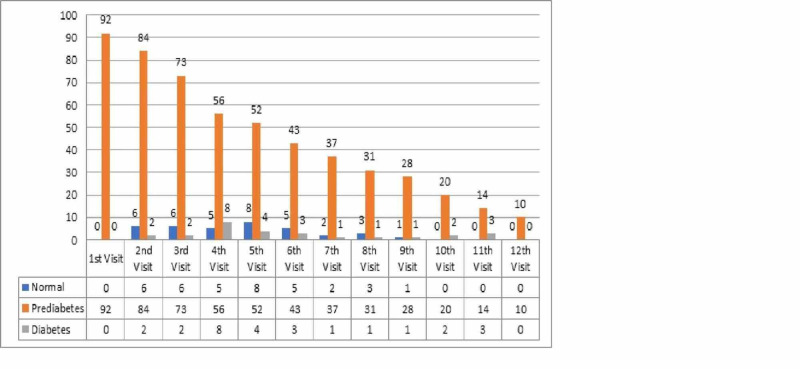
Frequencies and proportions presented indicate the number of follow-ups at each visit among prediabetics in different glycemic states The x-axis represents the number of visits and the y-axis represents the proportion of study participants at each follow-up visit.

﻿Figure 1 is a bar chart showing 92 prediabetic participants at the first visit. It presents the transformation to different glycemic states along with follow-up, with the median number of six visits for study participants and a median follow-up period of 44 months. Patients had various frequency of visits depending on many factors including the glycemic status and presence of other comorbidities as determined by the treating physician.

Table 2 shows the accomplishment of the objective of this study, which was to identify the prediabetes outcome after various interventions. The majority of the participants (76.08%) remained prediabetic, 16.30% regressed to NGS, and 7.6% progressed to diabetes. Age, gender, and initial BMI showed no significant differences in various glycemic groups, although it is worth noting that the age of individuals in diabetes group (41.71±9.3, mean ± SD) was less than that of NGS (48.2±12.89) and female with NGS twice higher than male. Furthermore, co-morbidities such as hypertension and dyslipidemia were not shown to be associated with either outcome. However, we found that 66.67% of patients who regressed to NGS had hypertension, which equals two-thirds of NGS patients. Also, we found that 86.67% of patients who regressed to NGS had dyslipidemia.

Regarding smoking, we found that two (13.33%) smokers, in contrast to 13 non-smokers (86.67%), regressed to NGS, while seven (100%) progressed to DM. Interestingly, about 64.13% took metformin; however, we found that only 16.9% of them regressed to NGS, while 76.3% remained at the prediabetic range and 6.8% progressed to diabetes. This showed that metformin produced no significant difference among the outcome groups. As expected, we found a significant difference between different glycemic groups in FBS and HbA1c at the end of follow-up, p<0.001.

**Table 2:** Comparison of various sociodemographic, clinical characteristics and comorbidity variables based on response across “glycemic range at last visit” Values are presented as mean ± standard deviation (SD) or numbers and percentages (%). Tests used: Fisher's exact test was used to determine associations between categorical variables, while the Kruskal-Wallis test was used for non-normally distributed continuous variables. BMI: body mass index, GDM: gestational diabetes mellitus, PCOS: polycystic ovarian syndrome, CVD: cardiovascular disease, FBS: fasting blood sugar, HbA1c: hemoglobin A1c, LDL: low-density lipoprotein cholesterol, HDL: high-density lipoprotein cholesterol. *Statistically significant at p<0.05.

Variable	Normoglycemia (N=15)	Prediabetes (N=70)	Diabetes (N=7)	P-Value
Age	48.2 ± 12.89	49±10.97	41.71 ± 9.3	0.203
Gender				0.329
Male	5 (33.33)	35 (50.00)	2 (28.57)	
Female	10 (66.67)	35 (50.00)	5 (71.43)	
BMI initial	33.38 ± 7.67	31.21±5.36	35.73 ± 5.89	0.113
Smoking				0.806
No	13 (86.67)	64 (91.43)	7 (100.00)	
Yes	2 (13.33)	6 (8.57)	0 (0.00)	
Hypertension				0.934
No	5 (33.33)	27 (38.57)	3 (42.86)	
Yes	10 (66.67)	43 (61.43)	4 (57.14)	
PCOS (women)				0.240
No	14 (93.33)	68 (97.14)	6 (85.71)	
Yes	1 (6.67)	2 (2.86)	1 (14.29)	
Dyslipidemia				0.188
No	2 (13.33)	11 (15.71)	3 (42.86)	
Yes	13 (86.67)	59 (84.29)	4 (57.14)	
CVD				1.000
No	14 (93.33)	63 (90.00)	7 (100.00)	
Yes	1 (6.67)	7 (10.00)	0 (0.00)	
Final visit				
FBS (mmol/L)	5.28 ± 0.35	5.94±0.59	7.76 ± 0.81	<0.001*
HbA1c (%)	5.51 ± 0.07	5.98±0.48	7.22 ± 0.37	<0.001*
LDL (mmol/L)	2.6 ± 0.66	2.76±0.96	2.78 ± 0.59	0.926
Triglyceride (mmol/L)	1.34 ± 0.73	1.29±0.54	1.55 ± 1	0.937
HDL (mmol/L)	1.11 ± 0.25	1.21±0.52	0.97 ± 0.19	0.142
Total-cholesterol (mmol/L)	4.3 ± 0.85	4.4±0.86	4.25 ± 0.52	0.813
Intervention:				
Metformin				0.931
No	5 (33.33)	25 (35.71)	3 (42.86)	
Yes	10 (66.67)	45 (64.29)	4 (57.14)	
Referral for the bariatric surgery				0.671
No	14 (93.33)	67 (95.71)	7 (100.00)	
Yes	1 (6.67)	3 (4.29)	0 (0.00)	
Referral to dietician				0.392
No	14 (93.33)	59 (84.29)	5 (71.43)	
Yes	1 (6.67)	11 (15.71)	2 (28.57)	

Table 3 shows the lipid profiles, glycemic indices, and BMI at the final visit. Sign and paired t-tests were used to assess the difference between the pre- and post-intervention groups. BMI showed an average increase of 0.4 kg/m2 from the baseline, while HbA1c showed a significant decrease, with p<0.001. Other parameters, including FBS, showed some improvement, though this was not significant, p >0.05.

**Table 3:** Overall comparison between pre- and post-continuous variable at baseline and the end of the follow-up Values are presented as mean ± standard deviation (SD) for HDL and median (IQR) for non-normally distributed data. Test used: Sign test for association between continuous variables with non-normally distributed and paired t-test for associations between continuous variables with normally distributed variables. BMI: body mass index, FBS: fasting blood sugar, HbA1c: hemoglobin A1c, LDL: low-density lipoprotein cholesterol, HDL: high-density lipoprotein cholesterol. *Statistically significant at p<0.05.

Variable	Initial visit	Last visit	p-value
BMI (kg/m^2^)	31.0(7.89)	31.4(7.28)	0.525
FBS (mmol/L)	5.9(0.65)	5.8(0.8)	0.050
HbA1c	6.1(0.3)	5.9(0.5)	0.001*
LDL (mmol/L)	2.7(1.23)	2.6(1.07)	0.428
Triglyceride (mmol/L)	1.2(0.77)	1.2(0.55)	0.114
HDL (mmol/L)	1.1±0.46	1.1±0.48	0.396
Total-cholesterol (mmol/L)	4.4(1.39)	4.2(1.06)	0.301

## Discussion

This study aimed to identify the prediabetes outcome in PHCs setting in Saudi Arabia. The study found that most of the patients, 84%, remained prediabetic or progressed to DM after three years of follow-up. This indicates a significant issue with prediabetics and their outcomes. This aligns with findings of Whitehall II cohort study in prediabetics defined by HbA1c, but not with FBS defined prediabetes, where it showed better reversion to normoglycemia [14]. However, our analysis lacks the comparison between prediabetics diagnosed based on FBS or HbA1c which in turn limits our ability to discuss which one of the tests had more reversion rates among prediabetics in the current study. In both studies, none of the patients were followed by OGTT, beyond pregnancy; OGTT has largely been eliminated from use in the diagnosis of diabetes, particularly due to the recent widespread standardization of the HbA1c assay and considering that our sample lacked any females with a GDM history [14]. Further studies among south Asian populations in 2015 demonstrated higher rates, with 58.9% of prediabetics progressing to DM; those higher rates can be attributed to the 10-year follow-up, as it is known that the yearly incidence of DM reaches up to 11% [15,16]. In the neighboring Gulf states, it reported that UAE which has a prevalence of DM comparable to that of Saudi Arabia, has an incident rate of DM 4.8 incident cases/1,000 person-years, other studies reported higher rates as 15.2 per 1000 person-years in overweight and obese individuals; all in retrospective studies [17,18].

This study shows that nearly 16% regressed to NGS. Similar studies were conducted in Shanghai to investigate regression to NGS with a 10-year follow-up. They showed that 22.5% regressed from prediabetes to NGS [19]. This finding was more prominent in those following healthier lifestyles, i.e., males who exercised more and females who had smaller waist circumferences, as it is known that certain behaviors and measures will affect the outcome of prediabetes [19].

Overall, our study found borderline significance in FBS and a significant difference in HbA1c, while BMI showed no difference from the baseline. By contrast, a 12-month post-intervention lifestyle modification among Arabs resulted in significant changes in body weight, BMI, and FBS [20]. Similar to our findings, interventional studies in Saudi Arabia showed a significant reduction in HbA1c in an even shorter, six-month duration [21]. HbA1c at the end of follow up was 5.8% ± 0.3% vs. 5.9% (0.5%) in the current study across glycemic groups and different interventions [21]. Moreover, a study on prediabetic overweight Japanese individuals confirmed those findings, showing the effectiveness of lifestyle modification in the prevention of DM progression [22]. Even so, other studies have established a link between lifestyle intervention and the reduction of serious complications in longer follow-ups of up to six years. This shows that lifestyle intervention in prediabetics is associated with a 47% reduction of the risk of developing retinopathy, as there was a decline in the incidence of DM in the first place [23].

It is worth noting that though initial BMI was 31.9±5.89 at the baseline, only a fraction of the cohort was referred to a dietician (15.22%), while 4.35% were referred to bariatric surgery. Among those, no data indicated whether they actually had the surgery or visited the dietician. Thus, we cannot reflect the outcome based on referral status alone. Of those who had documented referral to a dietician in our study shows, 7.14% experienced a remission to NGS. At the same time, the majority remained in the prediabetic range. These numbers reflect those who had been referred to a dietician and do not account for lifestyle advice given during the physician interview or elsewhere, as those data might not be attained from retrospective studies. Moreover, only two-thirds of those who regressed to NGS were on metformin, suggesting that the remaining third were undocumented or had no lifestyle intervention.

Medications play an important role in regression from prediabetic to NGS. The results of a diabetes prevention program show that lifestyle modifications and metformin significantly decrease the incidence of type 2 diabetes [9]. Also, an interventional study that included 120 prediabetic participants with hypertriglyceridemia showed that 70% of participants who had metformin treatment regressed to NGS [24]. In our study, which provides an insight on the actual setting of daily practices of PHCs in SA, the most commonly prescribed medication was metformin, which might be explained by the high initial BMI of the participants, as high BMI creates more of a risk of DM progression [8]. Only 16.9% of patients with metformin showed beneficial results and regressed to NGS, whilst the majority of patients on metformin remained in the prediabetic range. Of those who progressed to DM, 58% were on metformin. These observations might be a result of minimal medication compliance due to low perceived susceptibility or severity of the disease, as they have not developed full-blown diabetes yet. However, their adherence to metformin, perceived susceptibility, and severity were not assessed as a limitation of this study.

Gender was not a determinant factor for the outcome of this study. This supports the result of a study conducted in China, which found no association between gender and prognosis of a prediabetic state [19]. By contrast, a study conducted in ﻿Jeddah in 2016 found that ﻿DM has a strong association with a family history of diabetes, dyslipidemia in women, CVD in men, and hypertension [12]. Also, another study, conducted in Al-Kharj in SA, found that male gender, level of education, high BMI, marital status, and age above 45 had a significant predictive value with respect to prediabetes and diabetes as compared to NGS [13]. This study shows a notable relationship between obesity and prognosis. There is a higher initial BMI 35.73±5.89 for those who progressed to diabetes compared to those who did not, although that difference was not significant, p>0.05. Furthermore, there were no significant differences between age, dyslipidemia, hypertension, and CVD in terms of glycemic outcome. Our study cohort might not have been large enough to detect the difference, a larger sample size is needed to power the study. Predictors for progression, like advancing age, a family history of diabetes, 2-h PG, HbA1c, low HDL-cholesterol, and physical inactivity, have been observed in other studies [16]. Socioeconomic factors may impact the progression rates of prediabetic patients; developed countries are shown to have higher rates of progression to diabetes [11].

Although prediabetes can have devastating complications, we observed a steady reduction in the number of patients following up after the first follow-up visit, which might indicate below-optimal follow-up practices. According to the World Health Organization (WHO), blood glucose screening can treat or delay diabetes and its complications; therefore, we suggest the implementation of stringent policies to improve adherence to follow-up measures or the implementation of technology-assisted DPP and weight-loss interventions to minimize the incidence of diabetes [25,26].

This study faced several limitations that are worth mentioning. First, ﻿because this study used only EHRs to extract data, we found a relatively small sample size, which limits the generalization of our findings to only the capital city, Riyadh. However, we included all available prediabetic patients' data that fit the inclusion criteria. Also, this study represents the actual setting of prediabetes and the future transformation of health services in SA. A variety of possible factors-including first-degree-relative family history of DM, genetic susceptibility, physical activity, dietary intake, education, waist-to-hip ratio, and waist circumference-data were not documented as limitation for the current study and must be considered in future research [12,19]. Though most of the included study subjects were prescribed metformin in order to slow the progression of DM, adherence, and compliance could not be measured and this is attributed to the nature of the retrospective study; it also limits the extrapolation of our findings to other interventions like counseling and lifestyle modification.

 We encourage the physician to follow-up patients in a comprehensive approach, pay more attention to their BMI since the documented number of participants who were referred to dieticians was low, although, with a high BMI, it is better to meet with the diabetic physician and dietician simultaneously. We need more effort to make patients health-conscious, implement effective lifestyle changes to control body weight, and prevent progression to DM.

## Conclusions

In this study, we found that an overwhelming majority of participants remain in the prediabetic range or progress to DM after three years of follow-ups in with different interventions. A pharmacological intervention like metformin did not significantly affect the glycemic outcome in the most of patients though adherence could not be ascertained. Prospective studies with larger sample size are needed to assess the adherence and the outcome following lifestyle modifications or pharmacological intervention, along with new modalities like technology assisted interventions.
